# Impact of a microencapsulated *Enterococcus faecium*-phytogenic complex as an antibiotic alternative on growth performance, intestinal morphology, nutrient digestibility, antioxidant status and immune response in broilers

**DOI:** 10.1016/j.psj.2026.108061

**Published:** 2026-10-30

**Authors:** Maryam Sahraei, Reza Mahdavi, Soudabeh Moradi, Leili Jamshidi

**Affiliations:** Department of Animal Science, College of Agriculture and Natural Resources, Razi University, Kermanshah, 6715685438, Iran

**Keywords:** Essential oil, Chitosan, Probiotic, Antibody

## Abstract

This study evaluated the effects of a microencapsulated *Enterococcus faecium*-phytogenic complex (Thyme, Oregano, Ajwain, and *Enterococcus faecium*; TOA-EF) on 360 Ross 308 male broilers. Birds were randomly assigned to four treatments (6 replicates of 15 birds): a basal diet (NC), basal + Bacitracin (PC), and the basal diet supplemented with 0.75 or 1.5 g/kg of TOA-EF. Our results showed that while body weight gain remained unaffected (ANOVA-P > 0.05), both TOA-EF supplementation and the PC treatment significantly reduced feed intake compared with the NC group (ANOVA-P < 0.001), following a significant linear trend (P = 0.009). The feed conversion ratio was significantly improved (ANOVA-P = 0.002) in TOA-EF birds compared to the NC group, with the lowest FCR observed in the 0.75 g/kg TOA-EF group (1.59) and followed by the 1.5 g/kg TOA-EF (1.61), versus 1.70 in the NC group; however, the PC group did not differ significantly from the NC. While crypt depth and villus width remained unaffected (ANOVA-P > 0.05), TOA-EF supplementation significantly affected jejunal morphology, exhibiting a linear (P = 0.025) and quadratic (P = 0.028) increase in villus height (ANOVA-P = 0.034). The villus height to crypt depth ratio showed significant linear (P = 0.016) and quadratic (P = 0.021) responses, although the overall treatment effect was not significant (ANOVA-P = 0.062). Villus surface area showed a significant linear response to TOA-EF supplementation (ANOVA-P = 0.039, Linear-P = 0.043). Ileal digestibility of dry matter, ash, calcium, and phosphorus remained unaffected by dietary treatment (ANOVA-P > 0.05). Ileal CP digestibility differed significantly among dietary treatments (ANOVA-P = 0.001), with significant linear (P = 0.013) and quadratic (P < 0.0001) responses to TOA-EF supplementation. The highest mean CP digestibility (79.0%) was observed in the 1.5 g/kg group. Total antioxidant capacity, superoxide dismutase and glutathione peroxidase activities, and antibody titers against Newcastle disease virus remained unaffected (ANOVA-P > 0.05). The overall treatment effect on serum malondialdehyde concentrations was not significant (ANOVA-P = 0.236), although a significant linear response was detected (P = 0.045). Overall, TOA-EF supplementation influenced feed efficiency and selected aspects of intestinal morphology and protein digestibility, without affecting body weight gain and this blend may have potential as an alternative to antibiotic growth promoters in broiler diets.

## Introduction

Intensive poultry production systems impose significant physiological stress on broiler chickens. For decades, the industry has relied on sub-therapeutic antibiotics to promote growth and manage enteric health (Puvača et al., 2022). However, this practice has raised global concerns regarding antimicrobial resistance and pharmacological residues (Ahmad et al., 2022). Consequently, strict regulatory bans were implemented in regions like the European Union (Puvača et al., 2022). This shift catalyzed the search for sustainable alternatives, such as probiotics and phytogenic compounds, to maintain productivity and gastro-intestinal health (Ali et al., 2024; Banday et al., 2025). The escalating global urgency to eliminate pharmacological residues has promoted the adoption of antibiotic-free production models. Regulatory restrictions and consumer demand for responsible food production have encouraged this transition. To maintain intestinal homeostasis and mitigate metabolic dysbiosis, the industry is transitioning toward bioactive interventions. Functional alternatives, such as organic acids, probiotics, prebiotics and phytogenic complexes, may support intestinal health through different mechanisms (Ali et al., 2024). These substances may support broiler productivity by modulating intestinal health and nutrient utilization (Gurram et al., 2024).

Phytogenic feed additives have emerged as primary candidates for replacing synthetic promoters. Thyme (*Thymus vulgaris L.*) is a prominent herb characterized by high concentrations of thymol, carvacrol, p-cymene and γ-terpinene (Azaz et al., 2004; Saied et al., 2025). These compounds exhibit potent antimicrobial and antioxidant properties (Puvača et al., 2022). In broiler nutrition, thyme stimulates the secretion of endogenous digestive enzymes like amylase and protease (Lee et al., 2003; Meligy et al., 2023; Madkour et al., 2024; Saied et al., 2025). Studies also suggest that thyme oil can improve immune organ indices, contributing to a better overall physiological status (Saied et al., 2025). Oregano (*Origanum vulgare L.*) may disrupt the cell membranes of enteric pathogens and may influence oxidative stress and immune responses (Ruan et al., 2021; Puvača et al., 2022). Furthermore, oregano exhibits protective effects against necrotic enteritis (Jin et al., 2022). Ajwain (*Trachyspermum ammi*) is rich in essential oils that offer antimicrobial, germicidal, antifungal, and antispasmodic benefits (Shroha et al., 2018). Dietary inclusion suppresses *Escherichia coli* while promoting beneficial *Lactobacilli* (Valiollahi et al., 2014). *Enterococcus faecium* is a prominent lactic acid-producing bacterium ubiquitous in the avian digestive tract (Kochkina et al., 2020). This probiotic strain adheres to the intestinal mucosa and forms a protective biofilm. This layer regulates mucosal immunity and may prevents pathogens like *Salmonella* from colonizing the gut (Khalifa and Ibrahim, 2023).

While the individual benefits of these phytogenics and probiotics have been investigated as dietary supplements in broiler nutrition, their combined application represents a more advanced nutritional strategy. Combining diverse bioactive compounds may yield better biological outcomes than single-ingredient interventions. However, volatile essential oils and live microbial cells are sensitive to environmental conditions and harsh stomach acids (Zhang et al., 2024). Microencapsulation technology has been investigated as an approach to address the stability limitations of bioactive compounds during storage and gastrointestinal transit. Chitosan, a natural biopolymer with intrinsic antioxidant activity, can provide additional functional benefits by enhancing the protective effects of encapsulated compounds and potentially promoting intestinal epithelial integrity (Hosseini and Meimandipour, 2018; Nouri, 2019). Compared with conventional lipid-based coating systems, chitosan-based encapsulation matrices may offer improved protection against premature degradation and facilitate controlled release of active components along the gastrointestinal tract, thereby supporting optimal mucosal development and function (Hosseini and Meimandipour, 2018; Nouri, 2019). Consequently, this delivery strategy may improve the stability and bioavailability of encapsulated compounds, allowing their effective delivery to the small intestine and improving their potential beneficial effects on intestinal health and host performance. Therefore, we aimed to investigate the possible effects of this microencapsulated *Enterococcus faecium*-phytogenic complex as a natural replacement for antibiotic growth promoters on growth performance, intestinal morphology, nutrient digestibility, antioxidant status and immune system in broilers.

## Material and methods

### Experimental design, animals and diet

All experimental procedures received formal approval from the Animal Ethics Committee of the Department of Animal Science at Razi University, Iran. A total of 360 one-day-old Ross 308 male broiler chicks were randomly assigned to four dietary treatments, with six replicate pens per treatment and 15 birds per pen. The experimental groups comprised a negative control (NC), a positive control (PC) supplemented with 0.5 g/kg bacitracin methylene disalicylate, and two NC-based treatments fortified with either 0.75 g/kg or 1.5 g/kg of a microencapsulated *Enterococcus faecium*-phytogenic blend. The microencapsulated *Enterococcus faecium*-phytogenic complex used in this study, commercially known as Noviherb Plus, is a microencapsulated formulation containing essential oils from Oregano (*Origanum vulgare L.*), Ajwain (*Trachyspermum ammi*) and Thyme (*Thymus vulgaris L.*) each included at a concentration of 2%. This complex is fortified with the probiotic bacterium *Enterococcus faecium* at a high concentration of 1 × 10¹¹ CFU/g. The chemical composition of the essential oils from ajwain, thyme, and oregano was analyzed by GC-MS using an HP 7890 Network GC System coupled with a 5973 Network Mass Selective Detector (Agilent Technologies, California, USA). The oregano oil was predominantly composed of carvacrol (23.54%), γ-terpinene (20.50%), thymol (15.41%), and germacrene D-4-ol (9.26%). The major constituents of ajwain oil were thymol (54.32%), p-cymene (21.74%), γ-terpinene (19.38%), and carvacrol (0.42%). In thyme oil, the predominant compounds were thymol (44.35%), p-cymene (12.30%), carvacrol (8.10%), and γ-terpinene (6.80%). The microencapsulated blend was incorporated into the diets at 0.75 or 1.5 g/kg. Based on the product composition, each inclusion level supplied thyme, oregano, and ajwain essential oils at 150 and 300 mg/kg of feed, respectively, for each essential oil. The corresponding calculated concentrations of *Enterococcus faecium* were 75 × 10¹⁰ and 150 × 10¹⁰ CFU/kg of feed, respectively. To ensure the stability of the volatile bioactive compounds (primarily thymol and carvacrol) and to protect the probiotic cells from the harsh acidic environment of the upper gastrointestinal tract, the entire complex is microencapsulated using a natural chitosan polymer. This advanced delivery system utilizes a specialized spraying and cooling technique to create a protective barrier, facilitating a controlled and targeted release of the active ingredients within the small intestine. By protecting the core materials from premature oxidation and degradation, this microencapsulation technique optimizes nutrient digestibility and enhances the overall metabolic efficiency of the broiler chickens.

The experiment lasted for 42 days and was conducted in floor pens (1.4 × 1.5 m) with deep-litter in an environmentally controlled house. Birds were offered mash diets formulated according to the nutritional recommendations for Ross 308 broilers and had ad libitum access to feed and water. The ingredient composition and calculated nutrient contents of the experimental diets are shown in Table 1. The experiment lasted 42 days. The starter, grower, and finisher diets were provided from days 1-10, 11-28, and 29-42, respectively. The additives were added to the basal diets as a top-dressing.

**Table 1:** Ingredients and nutrient composition of the basal diets.

Ingredients (%)	Starter (1-10 days)	Grower (11-28 days)	Finisher (29-42 days)	Calculated nutrient composition			
Corn	52.30	60.42	62.18	ME (kcal/kg)	2950	3000	3070
Soybean meal	39.80	32.70	30.50	CP (%)	22.00	19.50	18.50
Soybean oil	3.10	2.80	3.6	Dig-Lys (%)	1.28	1.15	1.03
Dicalcium phosphate	2.30	1.80	1.7	Dig-Met+Cys (%)	0.95	0.87	0.80
Limestone	0.80	0.70	0.70	Dig-Thr (%)	0.86	0.77	0.69
L-Lys HCL	0.27	0.30	0.21	Ca (%)	0.96	0.82	0.77
DL-Met	0.38	0.35	0.30	Available P (%)	0.48	0.40	0.37
L-Thr	0.16	0.16	0.10				
Sodium bicarbonate	0.34	0.30	0.27				
Salt	0.18	0.17	0.18				
Mineral-Vitamin premix^1^	0.27	0.22	0.20				
Choline chloride	0.10	0.08	0.06				
	100	100	100				

1.Supplied per kilogram of diet: vitamin A, 9000 IU; vitamin D3, 2000 IU; vitamin E, 18 IU; vitamin B12, 0.15 mg; riboflavin, 6.6 mg; calcium pantothenate, 10 mg; niacin, 30 mg; choline, 500 mg; biotin, 0.1 mg; thiamine, 1.8 mg; pyridoxine, 3 mg; folic acid, 1 mg; vitamin K3, 2 mg; antioxidant (Ethoxyquin), 100 mg. zinc, 50 mg; manganese, 100 mg; copper, 10 mg; Fe, 50 mg; I, 1 mg; Se, 0.2 mg.

The additives were incorporated into the basal diet by top-dressing after premixing with ground soybean meal to ensure uniform distribution.

### Production performance

Growth performance parameters were determined throughout the 42-d experimental period. Body weight gain (BWG) was calculated for each replicate as the difference between final and initial collective body weights. Feed intake (FI) was calculated by subtracting the residual feed from the total feed supplied to each replicate. Mortality-adjusted FI was obtained by correcting feed consumption for birds that died during each experimental phase based on their body weight at the time of death. Feed conversion ratio (FCR) was calculated using mortality-corrected FI divided by BWG.

### Intestinal morphology

At the end of the 42-d experimental period, one bird per replicate (six birds per treatment) with a body weight closest to the replicate mean was selected for intestinal histomorphological evaluation. Following euthanasia by jugular venesection, a 2-cm segment of the jejunum was collected approximately 5 cm proximal to Meckel's diverticulum and immediately fixed in 10% neutral-buffered formalin. Tissue samples were dehydrated through a graded ethanol series, cleared in xylene, embedded in paraffin, sectioned at 5 μm using a rotary microtome (CUT4055, microTec Laborgeräte GmbH, Germany), and stained with hematoxylin and eosin. Histomorphological measurements, including villus height (VH; from the villus tip to the crypt base), villus width (VW; measured at the midpoint of the villus; and crypt depth), and crypt depth (CD; measured from the crypt opening to its base), were performed on digital images captured using an Olympus BX51TF light microscope (Olympus, Japan) equipped with a computer-assisted image analysis system. The villus-to-crypt ratio (VH/CD) was calculated by dividing VH by CD. Villus surface area (VSA) was calculated according to the method of Sakamoto et al. (2000) using the following equation: VSA = 2π × (VW/2) × VH (π = 3.14).

### Nutrient digestibility

Apparent ileal digestibility (AID) of dry matter (DM), crude protein (CP), ash, calcium (Ca), and phosphorus (P) was determined using titanium dioxide (TiO₂) as an indigestible marker. Seventy-two hours before the end of the experiment, experimental diets were supplemented with 0.5% (5 g/kg) titanium dioxide (TiO₂; Merck, Darmstadt, Germany) and fed to the birds. At the end of the trial, two birds per replicate were euthanized, and ileal digesta was collected from the segment extending from Meckel's diverticulum to 5 cm proximal to the ileocecal junction. Digesta samples from birds within the same replicate were pooled and stored at −20°C until analysis. Representative 1-kg samples of the experimental diets fed during the marker period were also collected for chemical analysis.

Before analysis, ileal digesta samples were freeze-dried and ground to obtain a homogeneous powder. Dry matter content of feed and digesta was determined by oven-drying at 105°C to constant weight (AOAC, 2005). CP concentration was determined by the Kjeldahl method (N × 6.25; AOAC, 2005). Ash content was measured by incineration in a muffle furnace at 600°C for 2 h. Ca concentration was determined by atomic absorption spectrophotometry (ZEEnit 700p, Analytik Jena, Germany), whereas total P was measured using the ammonium vanado-molybdate colorimetric method with a spectrophotometer (Unico S2100, UNICO, NJ, USA). Titanium dioxide concentrations in feed and digesta were determined according to the colorimetric method described by Short et al. (1996). AID coefficients were calculated using the index method according to the following equation:

$$AID_{nutrient}=1-\;\left\{\left(\frac{Ti_{Diet}}{Ti_{Digesta}}\right)\times \left(\frac{Nutrient{\;}_{Digesta}}{Nutrient{\;}_{Diet}}\right)\right\}$$

where $Ti_{Diet}$​ and $Ti_{Digesta}$ represent the titanium dioxide concentrations in the diet and ileal digesta, respectively, and $Nutrient{\;}_{Diet}$ and $Nutrient{\;}_{Digesta}$ represent the concentrations of the nutrient in the diet and ileal digesta, respectively.

### Antioxidant parameters and antibody titers against Newcastle disease virus

Humoral immune response was evaluated following vaccination against Newcastle disease virus (NDV). Birds received a live NDV vaccine (CEVAC® NEW L, La Sota strain, CEVA-Phylaxia, Hungary) via drinking water on day 30. Blood samples (2 mL) were collected from one identified bird per replicate on days 28 and 40. Samples collected on day 28 were used to determine baseline NDV antibody titers before vaccination and to confirm comparable humoral immune status among the experimental groups. The second blood sampling was performed on day 40 (10 days post-vaccination) to evaluate the antibody response induced by vaccination. Serum was separated by centrifugation at 3,000 rpm for 15 min and stored at −20°C until analysis. Antibody titers against NDV were determined using the standard hemagglutination inhibition (HI) assay.

At the end of the 42-d experimental period, one bird per replicate (six birds per treatment) with a body weight closest to the replicate mean was selected to assess antioxidant status. Two 4-mL blood samples were collected from each bird. One sample was transferred into an EDTA-coated tube for whole-blood analysis, whereas the second sample was collected into a plain tube (without anticoagulant) for serum preparation. Serum was separated by centrifugation and stored at −20°C until analysis. Serum malondialdehyde (MDA) concentration was determined according to the method of Ohkawa et al. (1979), based on the reaction with thiobarbituric acid followed by butanol extraction and spectrophotometric measurement. Superoxide dismutase (SOD) and glutathione peroxidase (GPx) activities were determined using commercial RANSOD and RANSEL assay kits (Randox Laboratories, Crumlin, UK), respectively, with GPx activity measured in whole blood. Serum total antioxidant capacity (TAC) was determined according to the method described by Mahmoud and Hijazi (2007). All measurements were performed spectrophotometrically according to the respective assay protocols.

### Statistical analysis

The pen was considered the experimental unit for all response variables, with six replicate pens per treatment. For growth performance, data were recorded at the pen level. For intestinal morphology, antioxidant parameters, and antibody titers against Newcastle disease virus, one representative bird was sampled from each pen, and the measurements obtained were considered representative of that pen. For ileal nutrient digestibility, digesta samples from two birds within each pen were pooled to form a single composite sample, which was analyzed as one experimental unit. Accordingly, six replicate observations per treatment were used for statistical analysis of each response variable.

Data were analyzed using the GLM procedure of SAS software (Version 9.4; SAS Institute Inc., Cary, NC, USA). Dietary treatment was included as the fixed effect. The pen was considered the experimental unit for growth performance variables, including BWG, FI and FCR. For jejunal histomorphology, antioxidant indices, NDV antibody titers and AID, the individual bird sampled from each replicate served as the experimental unit. When treatment effects were significant (P ≤ 0.05), means were separated using Duncan's multiple range test. Orthogonal polynomial contrasts were performed to evaluate linear and quadratic responses to increasing dietary inclusion levels of the microencapsulated *Enterococcus faecium*-phytogenic complex. Results are presented as means ± pooled standard error of the mean (SEM).

## Results

### Production performance

The effects of dietary treatments on the growth performance parameters of broiler chickens are summarized in Table 2. The results indicated that BWG was not significantly affected by TOA-EF or the antibiotic growth promoter (ANOVA-P > 0.05). In contrast, dietary treatments significantly affected FI (ANOVA-P < 0.0001). Birds supplemented with TOA-EF consumed significantly less feed than the NC group (4533 and 4543 g vs. 4837 g). Statistical analysis revealed a significant linear (P = 0.009) reduction in FI as supplementation levels increased, while no significant quadratic effect was detected (P = 0.956). Furthermore, the FCR was significantly improved by TOA-EF supplementation (ANOVA-P = 0.002). The lowest FCR (1.59) was achieved in the 0.75 g/kg group compared to the NC group (1.70).

**Table 2:** Effects of dietary inclusion of *Enterococcus faecium* -phytogenic blend on growth performance of broiler chicks.

Items		NC	PC	0.75 g/kg TOA-EF	1.5 g/kg TOA-EF		SEM		P-value		
									ANOVA	Linear	Quadratic
**BWG** (g; 1-42 days)		2855	2821	2860	2829		34.5		0.101	0.843	0.955
**FI** (g; 1-42 days)		4837 ^a^	4595 ^b^	4533 ^b^	4543 ^b^		27.6		<.0001	0.009	0.956
**FCR** (1-42 days)		1.70^a^	1.63 ^ab^	1.59 ^b^	1.61 ^b^		0.018		0.002	0.070	0.133

A significant difference was considered with P < 0.05. A tendency toward difference was considered with 0.05 < P < 0.10.

**NC**: basal diet without additives; **PC**: positive control (basal diet supplemented with 0.5 g/kg bacitracin methylene disalicylate); **TOA-EF**: basal diet supplemented with *Enterococcus faecium***-**Thyme-Oregano-Ajwain complex.

**BWG:** body weight gain, **FI:** feed intake, **FCR:** feed conversion ratio.

### Intestinal morphology

The effects of dietary treatments on the jejunal morphology parameters of broiler chickens are summarized in Table 3. The results indicated that VW and CD were not significantly affected by TOA-EF or the antibiotic growth promoter (ANOVA-P > 0.05). VH differed significantly among dietary treatments (ANOVA-P = 0.034). Mean VH was higher in the 0.75 and 1.5 g/kg TOA-EF groups (918 and 891 μm, respectively) than in the NC group (817 μm). Both linear (P = 0.025) and quadratic (P = 0.028) responses were significant. The overall effect of dietary treatment on the VH/CD ratio was not significant (ANOVA-P = 0.062). However, significant linear (P = 0.016) and quadratic (P = 0.021) responses were detected. VSA differed significantly among dietary treatments (ANOVA-P = 0.039). The highest mean VSA was observed in the 1.5 g/kg TOA-EF group (0.485 mm²), compared with 0.397 mm² in the NC group. A significant linear response was detected for VSA (P = 0.043), whereas the linear response in VW was not significant (P = 0.073).

**Table 3:** Effects of dietary inclusion of *Enterococcus faecium*-phytogenic blend on jejunal morphology of broiler chicks.

Items		NC	PC	0.75 g/kg TOA-EF	1.5 g/kg TOA-EF		SEM		P-value		
									ANOVA	Linear	Quadratic
VH (μm)		817^c^	804^c^	918^a^	891^b^		14.5		0.034	0.025	0.028
VW (μm)		158	164	166	173		2.53		0.524	0.073	0.201
CD (μm)		158	147	155	155		2.60		0.318	0.714	0.927
VH/CD		5.25	5.59	6.02	5.89		0.100		0.062	0.016	0.021
VSA (mm^2^)		0.397^b^	0.412^b^	0.480^a^	0.485^a^		0.018		0.039	0.043	0.106

A significant difference was considered with P < 0.05. A tendency toward difference was considered with 0.05 < P < 0.10.

**NC**: basal diet without additives; **PC**: positive control (basal diet supplemented with 0.5 g/kg bacitracin methylene disalicylate); **TOA-EF**: basal diet supplemented with *Enterococcus faecium***-**Thyme-Oregano-Ajwain complex.

**VH:** villus height, **VW:** villus width, **CD:** crypt depth, **VSA:** villus surface area.

### Nutrient digestibility

The effects of dietary treatments on the apparent ileal nutrient digestibility parameters of broiler chickens are summarized in Table 4. The results indicated that DM, ash, Ca, and P digestibilities were not significantly affected by TOA-EF or the antibiotic growth promoter (ANOVA-P > 0.05). In contrast, CP digestibility was significantly influenced by the experimental diets (ANOVA-P = 0.001). The 1.5 g/kg TOA-EF group had a mean CP digestibility of 79.0%, compared with 76.3% in the NC group and 74.4% in the PC group. Significant linear (P = 0.013) and quadratic (P < 0.0001) responses were observed.

**Table 4:** Effects of dietary inclusion of *Enterococcus faecium*-phytogenic blend apparent ileal nutrient digestibility of broiler chicks.

Items		NC	PC	0.75 g/kg TOA-EF	1.5 g/kg TOA-EF		SEM		P-value		
									ANOVA	Linear	Quadratic
**DM**		75.8	75.8	75.6	75.7		0.529		0.999	0.919	0.993
**CP**		76.3^b^	74.4^c^	76.0^b^	79.0^a^		0.470		0.001	0.013	<.0001
**Ash**		42.7	40.1	43.7	41.3		1.00		0.890	0.937	0.995
**Ca**		38.0	35.1	39.4	42.2		1.16		0.157	0.101	0.115
**P**		52.8	54.1	53.3	52.2		0.960		0.945	0.764	0.802

A significant difference was considered with P < 0.05. A tendency toward difference was considered with 0.05 < P < 0.10.

**NC**: basal diet without additives; **PC**: positive control (basal diet supplemented with 0.5 g/kg bacitracin methylene disalicylate); **TOA-EF**: basal diet supplemented with *Enterococcus faecium***-**Thyme-Oregano-Ajwain complex.

**DM:** dry matter, **CP:** crude protein, **Ca:** calcium, **P:** phosphorus.

### Antioxidant parameters and antibody titers against NDV

The effects of dietary treatments on serum biomarkers and antibody titers against NDV are summarized in Table 5. The results indicated that TAC, SOD, and GPX were not significantly affected by TOA-EF or the antibiotic growth promoter (ANOVA-P > 0.05), with no significant linear or quadratic responses observed for these parameters. For serum MDA, the overall effect of dietary treatment was not significant (ANOVA-P = 0.236). However, a significant linear response was detected across increasing TOA-EF inclusion levels (P = 0.045), whereas the quadratic response was not significant (P = 0.167). The lowest numerical MDA concentration was observed in the 1.5 g/kg TOA-EF group (1.54 mmol/mL), compared with 1.79 mmol/mL in the NC group. Antibody titers against NDV at 28 and 40 days of age did not differ significantly among dietary treatments (ANOVA-P > 0.05). The linear response in the change in antibody titers between the two sampling periods was not significant (P = 0.097).

**Table 5:** Effects of dietary inclusion of *Enterococcus faecium*-phytogenic blend on serum biomarkers and antibody against NDV of broiler chicks.

Items		NC	PC	0.75 g/kg TOA-EF	1.5 g/kg TOA-EF		SEM		P-value		
									ANOVA	Linear	Quadratic
TAC (mmol/l)		1.81	1.85	1.79	1.81		0.039		0.483	0.928	0.979
MDA (nmol/ml)		1.79	1.86	1.65	1.54		0.046		0.236	0.045	0.167
SOD (U/gHb)		2.40	2.57	2.45	2.60		0.052		0.574	0.246	0.445
GPX (U/gHb)		6.06	6.69	6.02	6.31		0.169		0.506	0.668	0.823
28 days		4.67	4.50	4.83	4.50		0.132		0.954	0.761	0.651
40 days (10 d post vaccination)		5.33	5.83	6.00	5.50		0.143		0.345	0.424	0.141
Difference		0.667	1.33	1.17	1.00		0.095		0.253	0.097	0.100

A significant difference was considered with P < 0.05. A tendency toward difference was considered with 0.05 < P < 0.10.

**NC**: basal diet without additives; **PC**: positive control (basal diet supplemented with 0.5 g/kg bacitracin methylene disalicylate); **TOA-EF**: basal diet supplemented with *Enterococcus faecium***-**Thyme-Oregano-Ajwain complex.

**TAC:** Total antioxidant capacity, **MDA:** Malondialdehyde, **SOD:** superoxide dismutase, **GPx:** glutathione peroxidase, **NDV** titers: Newcastle disease virus antibody titers.

## Discussion

Dietary supplementation with the TOA-EF complex significantly reduced FI and improved the FCR, while BWG remained unaffected. The reduction in FI alongside stable weight gain demonstrates that the birds achieved higher metabolic efficiency. Our results align with the observations of Amouei et al. (2021), El-Sayed et al. (2024) and Sahraei and Khalkhali-Evrigh (2026). Amouei et al. (2021) reported that thyme essential oil improved FCR by reducing total FI. Furthermore, El-Sayed et al. (2024) demonstrated that supplementing broiler diets with oregano led to a notable reduction in both FI and FCR. Sahraei and Khalkhali-Evrigh (2026) observed that birds receiving a combination of thyme and oregano essential oils exhibited the superior FCR compared to a control group. In the present study, the improvement in FCR was accompanied by changes in jejunal morphology and an increase in apparent ileal CP digestibility at the higher TOA-EF supplementation level. Jejunal VH and VSA increased in response to dietary supplementation (Table 3), while apparent ileal CP digestibility reached 79.0% in the 1.5 g/kg TOA-EF group, compared with 76.3% in the control group (Table 4). Greater villus height and surface area may indicate a larger anatomical area available for nutrient absorption, whereas the higher apparent ileal CP digestibility indicates improved apparent protein digestion at the higher supplementation level. These concurrent responses suggest that changes in intestinal morphology and protein digestibility may have contributed to the improved feed efficiency observed in this study.

Several mechanisms may help explain the observed improvements in feed efficiency. However, because the underlying physiological and biological mechanisms were not directly investigated in the present study, the following explanations should be regarded as potential mechanisms rather than demonstrated effects. Previous studies have suggested that phytogenic compounds may influence digestive enzyme activity (Lee et al., 2003; Meligy et al., 2023; Madkour et al., 2024; Saied et al., 2025), potentially contributing to improved nutrient digestion and utilization. In addition, thymol and carvacrol possess antimicrobial properties (Shroha et al., 2018; Puvača et al., 2022), suggesting that modulation of intestinal microbial populations could also contribute to the responses observed following phytogenic supplementation. The probiotic component of the TOA-EF complex may provide an additional influence on intestinal function and reduced the metabolic cost of subclinical immune activation, as suggested by previous research (Zhang et al., 2024). Furthermore, the chitosan microencapsulation probably ensured that these sensitive bioactive compounds reached the distal gut in a stable form, protecting them from early degradation in the upper gastrointestinal tract (Hosseini and Meimandipour, 2018, 2018; Nouri, 2019).

In contrast to our results, some researchers have reported significant increases in BWG following supplementation with thyme or oregano (Hassan et al., 2024; Banday et al., 2025; Saied et al., 2025). The lack of a weight gain response in our study might be attributed to the optimal health status of the flock, the specific nutrient density of the basal diet and environmental stressors.

VH, VH/CD and VSA exhibited both linear and quadratic increases in response to the supplementation levels if TOA-EF. Greater VH and surface area may provide a larger area for nutrient absorption and may be associated with improved intestinal function (Saied et al., 2025). In the present study, the morphological responses were accompanied by higher apparent ileal CP digestibility, which reached 79.0% in the 1.5 g/kg TOA-EF group. These findings suggest that changes in jejunal morphology may have contributed to improved nutrient utilization; however, a direct causal relationship between these responses cannot be established. Previous studies have also reported beneficial changes in intestinal morphology following dietary supplementation with phytogenic additives or related compounds (Malekzadeh et al., 2018; Kolbadinejad and Rezaeipour, 2020; Amer et al., 2021; He et al., 2021; Ghazanfari et al., 2023; El-Sayed et al., 2024; Mostafa et al., 2026). Several mechanisms may explain the morphological responses observed in the present study, although these explanations remain speculative because the underlying processes were not directly investigated. Thyme, oregano, and ajwain contain bioactive compounds with reported antimicrobial properties, which may influence the intestinal microbial environment and, in turn, help maintain mucosal integrity (Puvača et al., 2022). In addition, *Enterococcus faecium* may contribute to intestinal function through interactions with the gut microbiota and the intestinal mucosa (Zhang et al., 2024). Previous studies have suggested that microbial metabolites, including short-chain fatty acids, may support intestinal epithelial cell function (He et al., 2021; Khalifa and Ibrahim, 2023; Zhang et al., 2024). Phytogenic compounds have also been associated with effects on mucosal protection and inflammatory signaling pathways (Luettig et al., 2016; Puvača et al., 2022; Vaghela et al., 2025; Mostafa et al., 2026).

Our findings regarding the selective improvement of CP digestibility are consistent with recent research on phytogenic and probiotic additives. Malekzadeh et al. (2018) also documented improved nutrient utilization efficiency, reporting significant increases in the digestibility of organic matter. Gurram et al. (2024) reported a significant enhancement in protein retention following the use of various herbal and microbial supplements. Furthermore, Ali et al. (2026) observed that the combined methanolic extracts of thyme and moringa significantly improved CP digestibility without altering dry matter or ether extract utilization. Sahraei and Khalkhali-Evrigh (2026) also documented that birds receiving a combination of thyme, oregano, and probiotics exhibited higher protein digestibility compared to a control group. The improved protein utilization in our study was accompanied by increases in jejunal VH and VSA, suggesting that changes in intestinal morphology may have contributed to improved nutrient utilization. Several mechanisms may help explain the observed response, although the underlying processes were not directly investigated in this study. Phytogenic compounds have been proposed to influence digestive enzyme activity and gastrointestinal function, potentially contributing to nutrient digestion (Lee et al., 2003; Madkour et al., 2024; Saied et al., 2025). Meligy et al. (2023) also reported effects of encapsulated essential oils on the expression of genes associated with digestive processes. In addition, the antimicrobial properties of phytogenic compounds and the potential effects of *Enterococcus faecium* on the intestinal microbial environment may contribute to intestinal function and nutrient utilization (Puvača et al., 2022; Zhang et al., 2024; Ali et al., 2026). Previous studies have further suggested that phytogenic compounds may influence mucosal barrier function and the expression of genes associated with intestinal integrity (Malekzadeh et al., 2018; Ruan et al., 2021; Madkour et al., 2024; Vaghela et al., 2025; Park et al., 2025; Sahraei and Khalkhali-Evrigh, 2026). These proposed mechanisms provide possible explanations for the responses observed in the present study, but their specific contributions to the improvement in CP digestibility remain uncertain. In contrast to our results, some researchers have reported that herbal supplements do not always significantly influence systemic nutrient digestibility. For instance, Hernandez et al. (2004) did not detect significant changes in CP digestibility despite using plant extracts. The lack of response in those studies might be attributed to the free form of the additives used, which are often prone to rapid absorption or evaporation in the upper gastrointestinal tract. In our trial, the significant linear and quadratic response suggest that the higher bioavailability provided by the chitosan protection was a critical factor. While non-encapsulated forms may show inconsistent results.

Interestingly, apparent ileal CP digestibility was significantly lower in the PC group than in the NC group (74.4% vs. 76.3%). This difference may be related to treatment-induced changes in intestinal microbial ecology and nitrogen metabolism. Previous studies have shown that dietary bacitracin and other antibiotic growth promoters can alter intestinal microbial populations and gastrointestinal characteristics in broilers (Guban et al., 2006; Costa et al., 2017). Such alterations could potentially affect apparent ileal protein digestibility by modifying microbial nitrogen metabolism and the contribution of endogenous and microbial proteins to ileal digesta. Nevertheless, these mechanisms remain speculative because intestinal microbiota and endogenous nitrogen losses were not measured in the present study. Furthermore, the effects of antibiotic growth promoters on protein utilization are variable. Lu et al. (2020) reported improved apparent ileal amino acid digestibility in broilers fed avilamycin, whereas the effects of bacitracin methylene disalicylate on CP digestibility varied with sampling age and dietary conditions in the study by Gulizia et al. (2024). Therefore, the lower apparent ileal CP digestibility observed in the PC group should be interpreted as a treatment-related response under the conditions of the present experiment, rather than as evidence of a general negative effect of antibiotic supplementation on protein digestion.

Dietary supplementation with the TOA-EF complex did not significantly influence serum TAC, SOD, or GPx activities, although it resulted in a significant linear reduction in MDA concentrations. This pattern suggests that TOA-EF supplementation may have influenced lipid peroxidation without producing detectable changes in the measured antioxidant indices. Our findings regarding the reduction of lipid peroxidation are consistent with several previous reports. Ri et al. (2017) reported that oregano powder reduced lipid peroxidation in broilers, while Banday et al. (2025) observed lower serum MDA concentrations in birds supplemented with thyme powder. Similarly, Saied et al. (2025) reported reduced MDA concentrations following thyme oil supplementation. The observed reduction in MDA may be partly attributable to the antioxidant properties of bioactive compounds present in thyme, oregano, and ajwain. Certain phenolic compounds can donate hydrogen atoms to lipid peroxyl radicals, thereby interrupting the propagation of lipid oxidation (Banday et al., 2025). In addition, phytogenic compounds have been proposed to influence cellular antioxidant signaling, including the nuclear factor erythroid 2-related factor 2 (Nrf2) pathway (Ruan et al., 2021; Puvača et al., 2022). However, because Nrf2 signaling and related molecular responses were not evaluated in the present study, their involvement in the reduction in MDA remains a possible explanation rather than a demonstrated mechanism. The absence of significant changes in TAC, SOD, and GPx also indicates that the reduction in MDA was not accompanied by detectable changes in these measured antioxidant indices.

The absence of a significant effect of TOA-EF supplementation on the serum antibody response to NDV vaccination is consistent with findings from several previous studies. Malekzadeh et al. (2018) reported that hydroalcoholic extracts of different thyme species did not significantly affect antibody responses to ND vaccination. Similarly, Kolbadinejad and Rezaeipour (2020) found no significant effect of dietary ajwain seeds on NDV antibody titers in broilers, while Zaazaa et al. (2022) reported no significant differences in NDV antibody titers following supplementation with thyme and oregano essential oils. He et al. (2021) also found no significant differences in immune organ indices, including the thymus, spleen, and bursa of Fabricius, in broilers supplemented with *Enterococcus faecium*. Collectively, these findings suggest that phytogenic and probiotic additives do not consistently produce detectable changes in measured humoral immune responses or immune organ indices under the conditions examined. The lack of a significant antibody response in the present study indicates that TOA-EF supplementation did not measurably alter this specific humoral response, although it does not rule out potential effects on other aspects of immunity.

This study had a limitation regarding the potential contribution of chitosan to the observed responses. Because chitosan can have biological effects of its own, it may have influenced some of the measured parameters. However, its independent contribution could not be distinguished from the effects of the complete microencapsulated formulation. Therefore, the results should be interpreted cautiously, recognizing that the observed responses may reflect the combined effects of the formulation components, including chitosan.

## Conclusion

In conclusion, the microencapsulated *Enterococcus faecium*-phytogenic complex improved feed efficiency and selected indices of intestinal absorptive capacity and apparent ileal crude protein digestibility in broilers, without affecting body weight gain. These responses appeared to be primarily intestinal and metabolic rather than systemic, as most measured systemic antioxidant and humoral immune indices were not significantly affected. The findings suggest that this blend may serve as a plausible antibiotic alternative in broiler diets; however, further studies under diverse production conditions are needed to confirm its efficacy.

## Author contributions

**Maryam Sahraei,** contributed to the development of the experimental methods, conducted the animal trial and laboratory analyses, and contributed to the revision of the manuscript. **Reza Mahdavi, (Corresponding Author)** conducted the statistical analyses, interpreted the experimental data, organized the research data and materials, coordinated the research activities, and prepared and revised the manuscript. **Soudabeh Moradi,** conceived and designed the study, provided the necessary resources, supervised the research activities, coordinated the study, and critically revised the manuscript for important intellectual content. **Leili Jamshidi,** contributed to the experimental procedures, conducted data collection from the animal trial and laboratory analyses, and reviewed the manuscript.

All authors have read and approved the final version of the manuscript and agree to be personally and publicly responsible for all aspects of the work, ensuring that any questions regarding the accuracy or integrity of the research are thoroughly investigated and resolved.

## Disclosures

The authors declare no conflicts of interest.
